# Global Perfusion Practice Survey: Readiness of On-Call and Emergency Operation Rooms

**DOI:** 10.21470/1678-9741-2023-0236

**Published:** 2024-07-15

**Authors:** Salman Pervaiz Butt, Yasir Saleem, Nuno Raposo, Umer Darr, Gopal Bhatnagar

**Affiliations:** 1 Perfusion Department, Heart, Vascular and Thoracic Institute, Cleveland Clinic Abu Dhabi, Abu Dhabi, United Arab Emirates; 2 Department of Cardiothoracic and Vascular Surgery, All India Institute of Medical Sciences, Rishikesh, Uttarakhand, India; 3 Cleveland Clinic Abu Dhabi, Abu Dhabi, United Arab Emirates

**Keywords:** Emergencies, Reaction Time, Sample Size, Perfusion, Cardiac Surgical Procedures, Anesthetists, Patient Care, Delivery of Health Care

## Abstract

**Introduction:**

Perfusion safety in cardiac surgery is vital, and this survey explores perfusion practices, perspectives, and challenges related to it. Specifically, it examines the readiness of on-call and emergency operation rooms for perfusion-related procedures during urgent situations. The aim is to identify gaps and enhance perfusion safety protocols, ultimately improving patient care.

**Methods:**

This was a preliminary survey conducted as an initial exploration before committing to a comprehensive study. The sample size was primarily determined based on a one-month time frame. The survey collected data from 236 healthcare professionals, including cardiac surgeons, perfusionists, and anesthetists, using an online platform. Ethical considerations ensured participant anonymity and voluntary participation. The survey comprised multiple-choice and open-ended questions to gather quantitative and qualitative data.

**Results:**

The survey found that 53% preferred a dry circuit ready for emergencies, 19.9% preferred primed circuits, and 19.1% chose not to have a ready pump at all. Various reasons influenced these choices, including caseload variations, response times, historical practices, surgeon preferences, and backup perfusionist availability. Infection risk, concerns about error, and team dynamics were additional factors affecting circuit readiness.

**Conclusion:**

This survey sheds light on current perfusion practices and challenges, emphasizing the importance of standardized protocols in regards to readiness of on-call and emergency operation rooms. It provides valuable insights for advancing perfusion safety and patient care while contributing to the existing literature on the subject.

## INTRODUCTION

Several studies collectively underscored the paramount importance of perfusion safety in the context of cardiac surgery and cardiopulmonary bypass (CPB) procedures. Kurusz’s work highlighted that perfusion safety is an evolving field, necessitating constant adaptation to new safety measures and practices to align with advancements in medical technology and surgical techniques. Mulholland’s perspective underscored the proactive approach required to address perfusion safety issues, stressing the need for healthcare systems and practitioners to anticipate and prepare for future challenges. Similarly, Baker and Willcox’s 2006 survey offers vital data for informed enhancements in equipment and monitoring practices, enabling healthcare professionals to make evidence-based decisions to improve patient safety. Furthermore, Stammers and Mejak’s study shed light on the influence of perfusion practices on incident rates, allowing practitioners to tailor approaches for minimizing risks^[1,2,3,4]^.

Perfusion safety encompasses a range of practices, protocols, and measures designed to safeguard patients undergoing cardiac surgery procedures^[1,2]^. These procedures involve complex technologies, equipment, and coordination among healthcare professionals to maintain optimal blood flow, oxygenation, and temperature control.

Hence, by assessing the current understanding and practices related to perfusion safety^[5,6]^, this survey aims to gain insights into healthcare professionals’ perspectives, experiences, and challenges in this field. Understanding the factors influencing perfusion safety is essential for improving patient outcomes and reducing adverse events. Keeping that in mind, we have conducted a survey to look at Global Perfusion Practice regarding the readiness of on-call and emergency operation rooms from perfusion perspective.

We seek to understand the preparedness of operation rooms for perfusion procedures during urgent and emergency situations, where timely intervention is critical. Through the survey, we hope to identify potential gaps or areas for improvement in the readiness of operation rooms, equipment availability, and adherence to protocols^[7]^. Additionally, we aim to gain a better understanding of the perspectives and experiences of healthcare professionals involved in perfusion during emergency situations. The data collected from this survey will provide valuable information that can be used to enhance perfusion safety protocols, develop guidelines^[8]^, and improve overall patient care. By identifying best practices and potential challenges, we can work towards minimizing risks, reducing adverse events, and optimizing patient outcomes in emergency and on-call situations.

## METHODS

This was a preliminary survey conducted as an initial exploration before committing to a comprehensive study and there wasn't a predetermined sample size. The sample size was primarily determined based on a one-month time frame. This is a limitation to the study. The survey was designed to gather information about the readiness of on-call and emergency operation rooms from a perfusion perspective. The survey questions were carefully developed to assess one aspect of perfusion safety of having CPB circuit ready. The survey was designed to be simple yet concise, ensuring that participants could provide relevant insights without feeling overwhelmed. The target population for this survey included the cardiothoracic team, comprising cardiac surgeons, perfusionists, and anesthetists. Our goal was to capture a broader perspective by including healthcare professionals from different regions and healthcare settings.

The data for this survey were collected through an online survey platform. Participants were provided with a unique survey link and were able to access and complete the survey at their convenience. The online survey format allowed for efficient data collection and ensured the anonymity of participants. The survey included a multiple-choice question and open-ended questions to gather both quantitative and qualitative data.

### Ethical Considerations

In conducting this survey, ethical considerations were paramount. Anonymity and confidentiality were upheld, with no personally identifiable information collected or disclosed. Participation was voluntary, ensuring no coercion. Data collected was used solely for research purposes, maintaining privacy.

## RESULTS

The "Global Perfusion Practice Survey: Readiness of On-Call and Emergency Operation Rooms" was conducted over a period of one week, yielding a total of 236 responses. The survey consisted of six multiple-choice questions, and the distribution of responses for each question has been visually represented in the accompanying pie charts below. These charts provide a clear snapshot of the participants'choices and opinions regarding the readiness of on-call and emergency operation rooms from a perfusion perspective.

### Survey Questions

1. Your departmental perfusion practice for on-call emergency operation room/theatre readiness.
2. Why primed circuit?
3. Why dry circuit, why not primed?
4. Why no CPB circuit? (no dry or primed)
5. Individual's preference?
6. Your country of practice?

### Question 1

**Fig. 1:**
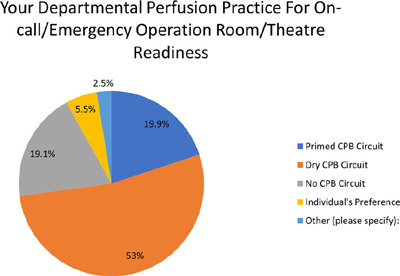
Question 1. CPB=cardiopulmonary bypass.

Out of the 236 responses received, it was found that the majority of participants (53%) opted to keep a dry circuit for emergencies during the night, while 19.9% had primed circuits readily available. On the other hand, 19.1 % indicated that they did not have a pump ready, and 2.5% selected an alternative option as shown in (Figure 1).

When exploring the "other" category, several interesting reasons were provided. Some participants mentioned that their practice focused on pediatric cases, where circuits needed to be tailored to the size of the patient. This uncertainty about the patient's size could lead to wastage of circuits if kept pre-primed. Another participant mentioned having two different sizes of primed extracorporeal membrane oxygenation circuits available, which could be utilized in real emergency situations, potentially eliminating the need for a primed heart-lung machine.

Additional insights revealed center-specific variations based on caseloads, types of cases performed at specific times, whether the center was a transplant referral center or dissection referral center, and the availability of surgeons. These responses highlight the diverse practices and considerations within perfusion settings, emphasizing the importance of center-specific factors and individual preferences when it comes to the readiness of on-call and emergency operation rooms.

### Question 2

The study done by Schulz S et al.^[9]^ has shown that wet-CPB circuit can be used safely after 72 hours of standby under regular clinical conditions.

**Fig. 2:**
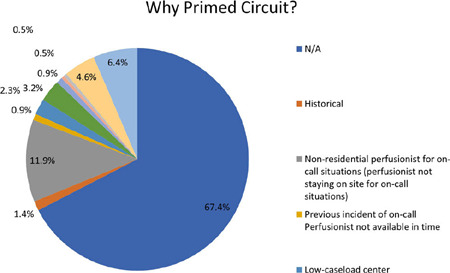
Question 2.

Out of the 218 respondents to Question 2, 147 individuals (67.4%) selected “Not applicable”. However, several reasons were provided by those who did keep a circuit primed. The most significant reason, mentioned by 26 respondents (11.9%), was the non-residential on-call situation. When perfusionists have longer response times and are not on-site, having a pump ready can be beneficial due to the extended time it takes to arrive at the hospital. One participant selected “other”, possibly indicating that they were not staying on-site during the on-call period. The (Figure 2) given below shows the percentage distribution of the question.

Three participants mentioned historical reasons for keeping a pump primed, although this rationale did not consider their current needs. The question arises as to whether this approach aligns with best practices. Two participants mentioned incidents where the perfusionist was not available in time, leading them to prime the pump as a precautionary measure to minimize the risk of recurrence.

Surgeons’ preferences were mentioned by seven respondents as a reason for pump readiness. However, one could argue that the decision to prime the pump should ultimately lie with the perfusionist, as they are responsible for priming and utilizing it when necessary. Ten participants indicated that a primed pump was kept due to the unavailability of an N+1 perfusionist. While a primed pump may enhance readiness in such cases, it raises the question of whether relying solely on a primed pump negates the need for a backup perfusionist — a critical consideration that warrants further examination.

Two participants mentioned working in transplant centers, while five mentioned working in low-caseload centers, and one mentioned covering multiple sites. The question of whether busy centers would benefit from a primed pump arises. Fourteen percent of respondents selected “other” and provided various reasons, including the absence of an N+1 perfusionist, previous incidents, prioritizing patient safety, operating in high-throughput centers, mitigating risks, and reducing time to initiate bypass in catastrophic events like stabbings. Different geographical areas may also have their own regulations; for instance, London has a one-hour response time. One respondent highlighted the challenges posed by response time, leading them to keep a pump ready as primed circuits improve response time and enhance safety.

Additionally, nine other valid selections provided reasons, including fatigue from previous cases, the lack of regular pump priming practices, and the presence of locum perfusionists who may not be quick enough to prime in emergencies when working at a new center. These factors highlight the complexity and individual considerations that influence the decision to prime pumps during on-call situations.

### Question 3

**Fig. 3:**
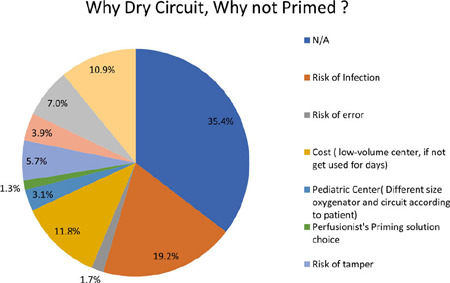
Question 3.

Among the respondents to Question 3, the primary reason for keeping a dry circuit, as reported by 44 individuals (19.2%), was the concern of infection risk as shown in (Figure 3). Further exploration is needed to understand the nature of this risk. Is it because they are unsure about the person who built and primed the pump or is it due to the potential risk of infection if a primed circuit is not used in a timely manner? Four respondents mentioned the risk of error, while 27 individuals stated that working in a low-caseload center would result in the pump not being used in time, rendering the circuits wasteful.

Seven participants mentioned working in a large institute, implying that varying perfusionists may have different preferences for priming pumps, leading them to refrain from priming to avoid potential conflicts. Thirteen respondents expressed concerns about tampering, raising questions about whether these concerns stem from colleagues, members of the theatre team, or other perfusionists. If such risks are compromising patient safety, they warrant further investigation, and steps should be taken to address toxic relationships within hospitals.

Additional nine participants mentioned avoiding issues related to improper priming or uncertainty about the prime, indicating that team dynamics and relationships might impact how perfusionists prepare their pumps for on-call situations. Sixteen individuals mentioned that they preferred to prime their pumps, and exploring the underlying reasons behind this preference, such as team dynamics, could shed light on potential underlying issues.

Twenty-five respondents mentioned other reasons, with some indicating that priming can be easily done in a hurry and does not take much time. One participant mentioned the need to sign off on each drug, highlighting a lack of trust or uncertainty about the contents of a primed circuit. Infection control was mentioned again, with concerns that an open reservoir would become unsterile after a few hours. One participant noted that a wet oxygenator’s oxygenation ability would degrade if left primed for too long. Another person mentioned the ease of circuit customization when the circuit is dry.

Manufacturers typically guarantee the oxygenator for six hours when primed. Many respondents indicated that multiple reasons applied to their decision. Transforming this question into a multiple-entry format and consolidating responses from the “other” category could provide a more comprehensive understanding of the participants’ perspectives. Among the 25 respondents who selected “other”, 22 described their reasons, which could offer valuable insights into additional factors influencing the decision to keep a dry circuit.

### Question 4

**Fig. 4:**
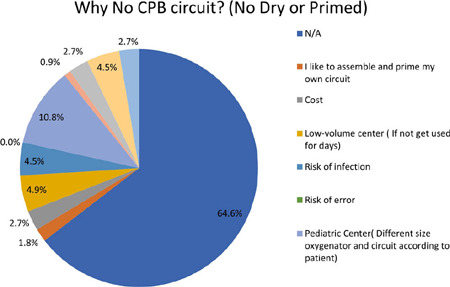
Question 4.

Among the respondents, the most significant reason for not having any type of circuit (dry or primed) ready was attributed to working in pediatric centers and the need for varying oxygenators, accounting for 10.8% of the responses as represented in (Figure 4). Two individuals who also worked in pediatric centers selected “other” without specifying their reasons. This aspect is closely related to the challenges faced in pediatric settings, where specialized equipment and customization are often required.

Additionally, 2.7% of respondents mentioned cost as a prohibiting factor, indicating that circuits are not prepared due to financial considerations. Low-volume centers accounted for 4.9%, suggesting that these centers may have fewer cases, making it less necessary to keep circuits ready at all times.

For some respondents, having a residential on-call arrangement allowed ample time to build and prime circuits if needed, representing 4.5% of the responses. The same percentage of respondents also cited infection risk as a deterrent to keeping circuits ready, reflecting the ongoing concern for maintaining a sterile environment.

Interestingly, one participant mentioned being dictated by the Louisiana Department of Health, although the specific details or requirements were not provided. This indicates that regulatory or institutional factors may influence the decision-making process regarding circuit readiness.

Overall, these responses highlight the diverse reasons why some individuals choose not to have circuits ready, including the specific requirements of pediatric centers, financial considerations, lowcase volumes, availability of time, infection risk concerns, and external regulatory factors. Understanding these factors is crucial for optimizing perfusion practices and ensuring patient safety.

### Question 5

**Fig. 5:**
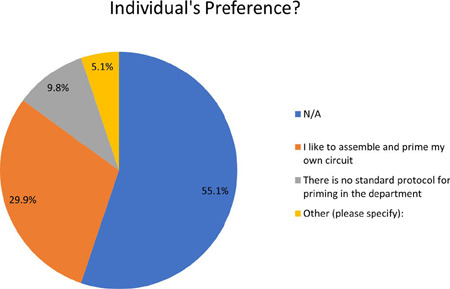
Question 5.

In terms of individual preferences, 29.9% respondents expressed a preference for assembling and priming their pumps (Figure 5), indicating a sense of ownership and control over the process. On the other hand, 21 individuals mentioned that there is no standard practice within their department, suggesting a lack of consistency or established protocols. It is worth considering whether it is fair to expect a perfusionist dealing with an emergency to build the circuit hastily, as one person noted a desire for a primed pump but stated that it is not practiced in their department.

Among those who opt for primed pumps, one respondent emphasized the importance of writing an expiration date on the pump, indicating a commitment to ensuring the freshness and reliability of the components. Another respondent mentioned that the person managing the emergency situation must perform a final check on the pump. In contrast, one person highlighted that the pumps are built by one perfusionist for another and are subject to verification by a second perfusionist, suggesting a collaborative and double-checking approach to pump assembly and readiness. These perspectives shed light on the varying practices and considerations regarding pump assembly and verification. The presence of individual preferences, lack of standardization, and differing approaches to quality assurance highlight the need for further discussion and development of best practices to ensure consistent and safe pump preparation in emergency situations.

### Question 6

**Fig. 6:**
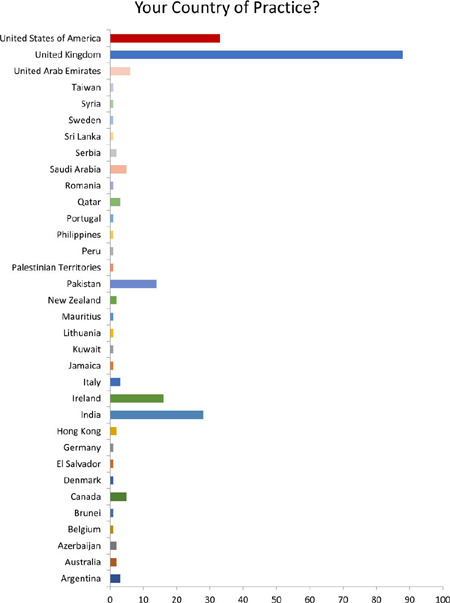
Question 6.

Among the countries represented in the survey, the top three countries with the highest number of respondents were the United Kingdom, United States of America, and India (Figure 6).

These countries displayed a notable presence in terms of survey participation. It is important to note that only countries with at least one response were included in the analysis, and therefore, the rankings may not reflect the overall global distribution of respondents.

## DISCUSSION

### Focus Analysis on United Kingdom & Ireland Subgroup

Since the majority of respondents (104 out of 236) in the survey were from the United Kingdom and Ireland, a subgroup (Table 1) was established to delve deeper into the analysis of perfusion practices concerning readiness in on-call and emergency room situations. During the data collection period, there were a total of 458 registered perfusionists and 54 cardiac units/hospitals in the United Kingdom and Ireland. Hence, this substantial dataset reflects the comprehensive representation of United Kingdom-based perfusionists, underlining the robustness of the data pool. United Kingdom subgroup data analysis of departmental perfusion practices for on-call emergency operation room/theatre readiness reveals a diversified landscape of approaches. Notably, a significant portion of respondents, comprising 52.9% (Table 2), prefer to utilize dry CPB circuits. This approach, devoid of any priming with blood or other solutions, could be attributed to several reasons. Firstly, concerns about infection risk, as indicated by 16.3% of respondents (Table 3), weigh heavily in favor of keeping circuits dry. Additionally, the avoidance of issues related to the priming process, as noted by 6.7% of respondents, underlines the importance of minimizing potential complications during critical procedures. Interestingly, 5.8% of respondents express a personal preference for priming their own circuits, suggesting that individual perfusionists’ practices play a role in this choice. In low-volume centers, cost considerations (4.8%) might drive the decision to opt for dry circuits. Moreover, the availability of sufficient time to prime in an emergency (3.8%) and considerations related to the viability of the oxygenator (2.9%) are also factors into the decision-making process. In summary, the prevalence of dry CPB circuits appears to stem from a combination of concerns related to infection, issues avoidance, personal preferences, and cost-effectiveness.

**Table 1:** United Kingdom and Ireland.

Response	Count	%
United Kingdom	88	84.6%
Ireland	16	15.4%
**Total**	**104**	**100.0%**

**Table 2:** Your departmental perfusion practice for on-call emergency operation room/theatre readiness (United Kingdom and Ireland subgroup).

Response	Count	%
Dry CPB circuit	55	52.9%
Primed CPB circuit	31	29.8%
No CPB circuit	13	12.5%
Individual’s preference	3	2.9%
Two primed ECMO of different sizes	1	1.0%
Cell saver setup	1	1.0%
**Total**	**104**	**100.0%**

CPB=cardiopulmonary bypass; ECMO=extracorporeal membrane oxygenation

**Table 3:** Why dry circuit, why not primed? (United Kingdom and Ireland subgroup).

Response	Count	%
N/A	44	42.3%
Risk of infection	17	16.3%
Risk of tamper	9	8.7%
Avoid issues (like, it was not primed properly or what was the prime)	7	6.7%
I like to prime my own circuit	6	5.8%
Multiple	5	4.8%
Cost (low-volume center, if not get used for days)	5	4.8%
Enough time to prime in an emergency	4	3.8%
Viability of oxygenator	3	2.9%
Risk of error	2	1.7%
Pediatric center (different size oxygenator and circuit according to patient)	1	1.0%
Historical	1	1.0%
**Total**	**104**	**100.0%**

Conversely, approximately 29.8% of respondents favor the use of primed CPB circuits. Factors influencing the choice of primed circuits include scenarios where perfusionists do not stay on-site for on-call duties (16.3%), situations where there is only one perfusionist on-call without an additional backup (N+1, 5.8%), and historical practices (1.9%) (Table 4). Transplant centers (1.9%) also lean towards primed circuits, likely due to the imperative for rapid readiness in such specialized environments. The data suggests that a range of factors, including staffing, historical practices, and specialized clinical needs, contribute to the adoption of primed CPB circuits. In contrast, 12.5% of respondents indicated that they do not keep any CPB circuit, whether dry or primed, for on-call emergencies. Factors influencing the choice is diverse, in pediatric centers, where different-sized oxygenators and circuits are required for each patient, not using any preassembled circuit is a prevalent practice (11.5%) (Table 5). The remaining 1.0% of respondents cited diverse reasons, including personal preference for circuit assembly, concerns about infection risk, and the absence of any historical issues in not having a circuit readily available.

**Table 4:** Why primed circuit? (United Kingdom and Ireland subgroup).

Response	Count	%
N/A	68	65.4%
Non-residential perfusionist for on-call situations (perfusionist not staying on site for on-call situations)	17	16.3%
One perfusionist for on-call situations, no N+1 for on-call situations	6	5.8%
No response	4	3.8%
Multiple reasons	4	3.8%
Transplant center	2	1.9%
Historical	2	1.9%
High throughput center. Transplantation, trauma, etc.	1	1.0%
**Total**	**104**	**100.0%**

**Table 5:** Why no CPB circuit? (no dry or primed) (United Kingdom and Ireland subgroup).

Response	Count	%
N/A	81	77.9%
Pediatric center (different size oxygenator and circuit according to patient)	12	11.5%
Risk of error	5	4.8%
I like to assemble and prime my own circuit	1	1.0%
Multiple	1	1.0%
No technical reason to do so	1	1.0%
Risk of infection	1	1.0%
Never been an issue to have a circuit ready	1	1.0%
Avoid issues	1	1.0%
**Total**	**104**	**100.0%**

Furthermore, individual preferences play a role in these practices, with 1% of respondents expressing a liking for either dry or primed circuits. Additionally, 21.2% of respondents indicated a preference for assembling and priming their own circuits, emphasizing the importance of individual autonomy and comfort in the perfusion process (Table 6). Various other responses, including the lack of a standard protocol, adapting practices to individual centers’ needs, and team-based approaches with safety checks, further underline the dynamic nature of perfusion practices and the influence of departmental cultures and protocols.

**Table 6:** Individual’s preference? (United Kingdom and Ireland subgroup).

Response	Count	%
N/A	64	61.5%
I like to assemble and prime my own circuit	22	21.2%
Risk of error	12	11.5%
I’d prefer a primed pump, but this is not done in my department	1	1.0%
Anyone can assemble and prime the circuit but person running the case must do safety check	1	1.0%
There is no standard protocol for priming in the department	1	1.0%
Select what is most suitable and safer to have at each individual centre	1	1.0%
Dry built or primed, happy for either	1	1.0%
Team members build pumps for others usage, mostly prime own circuits for cases using the standard protocol for priming. Circuit visually checked by second team member.	1	1.0%
**Total**	**104**	**100.0%**

In summary, this comprehensive analysis of this subgroup data highlights the intricate interplay of factors influencing perfusion practices in emergency operation room/theatre readiness. These factors encompass concerns about infection, staffing situations, cost-effectiveness, personal preferences, and specialized clinical needs. To optimize patient safety and efficiency, it is imperative for healthcare institutions to consider these diverse factors when developing standardized protocols and guidelines for perfusion practices in critical care settings.

### Comparison between Western and Non-Western Countries

When comparing data between Western and non-Western groups, we observe both similarities and differences in the practices related to the readiness of on-call and emergency on-call rooms.

### Similarities

**Preference for Primed CPB Circuits:** In both Western and non-Western countries, perfusionists prefer primed CPB circuits. The reasons for this preference include emergency readiness, previous incidents of delays, and ensuring that the pump is ready for immediate use.

**Concerns About Risk of Infection:** Both groups express concerns about the risk of infection associated with using pre-primed circuits, which can lead to a preference for dry circuits in some cases.

**Individual’s Preference:** The data shows that individual perfusionists’ preferences play a significant role in the choice between primed, dry, or no CPB circuits, irrespective of the country. Some perfusionists prefer to assemble and prime their own circuits.

**Variability in Protocols:** In both Western and non-Western countries, there is variability in departmental protocols related to priming CPB circuits, with some departments lacking standard protocols.

### Differences

**Use of Dry CPB Circuits:** Dry CPB circuits are more commonly preferred in Western countries, particularly in the United States of America. This is often due to cost considerations and the availability of resources, as well as a low caseload in some centers.

**Use of Primed CPB Circuits:** Primed CPB circuits are more frequently preferred in Western countries, such as the United Kingdom, to ensure readiness for emergency cases. This practice is less common in non-Western countries.

**No CPB Circuits:** Some countries, like Argentina and Jamaica, indicate having no CPB circuits ready, particularly in pediatric centers, which may be due to the availability of different-sized oxygenators and circuits.

**Departmental Policy:** Western countries, especially the United Kingdom, often prioritize departmental readiness for emergency cases, leading to a preference for primed circuits. In contrast, non-Western countries may rely more on individual perfusionists’ preferences.

**Cost Considerations:** Cost considerations, particularly related to low-volume centers and the potential for unused primed circuits being wasted, are more frequently cited in Western countries as a reason for using dry CPB circuits.

**Residential Perfusionists:** Some Western countries mention having residential perfusionists on-site for on-call duties, allowing for more flexibility in circuit preparation and quick response.

**Surgical Preference:** Surgical preference appears to be a more common factor influencing the choice of circuit in non-Western countries, such as India and Pakistan.

**Regulatory Influence:** Some non-Western countries mention that their circuit priming practices are influenced by local regulatory bodies or departmental decisions.

### Implications and Applications

The survey results have important implications and applications for perfusion safety. Practically, the findings highlight the need for standardized protocols and guidelines in on-call and emergency situations, addressing issues such as circuit priming. By establishing clear procedures, response time and patient outcomes can be improved. The survey also brings attention to concerns regarding infection risk, tampering, and error, emphasizing the importance of enhanced training, communication, and quality control measures. The results can be applied in various ways, serving as a benchmark for evaluating current practices, informing the development of standardized guidelines, and guiding further research in areas such as infection control and team dynamics. Based on the survey outcomes, recommendations include implementing standardized protocols, promoting collaboration among stakeholders, enhancing training programs, and conducting further research to improve perfusion safety practices. Overall, the survey provides valuable insights to drive evidence-based decision-making and enhance patient safety in perfusion.

### Limitations

The survey has certain limitations that need to be acknowledged. The sample size, based on voluntary participation, may not fully represent the entire perfusion community, leading to potential selection bias. Additionally, the survey’s reliance on multiple-choice questions may limit the depth of responses and overlook important nuances. Furthermore, the survey’s focus on the readiness of on-call and emergency operation rooms narrows the scope of the insights obtained. To address these limitations, future research should consider more comprehensive sampling methods to ensure a representative sample of perfusion professionals. Incorporating open-ended questions and qualitative interviews can provide a deeper understanding of experiences and challenges. Moreover, exploring other aspects of perfusion safety, such as infection control, equipment maintenance, and team communication, would provide a more holistic view. Future investigations should also examine the specific factors contributing to infection risk, the impact of team dynamics on safety, and the implications of different perfusion practices in diverse healthcare settings. By addressing these areas, future research can advance perfusion safety practices and enhance patient care.

## CONCLUSION

In conclusion, the survey on the readiness of on-call and emergency operation rooms in perfusion practice provides valuable insights into current practices and challenges in the field. The findings reveal the prevalence of keeping a dry circuit for emergencies during the night and the availability of pumps, while also highlighting factors such as non-residential on-call status, historical reasons, and surgeon preferences influencing these practices. Concerns regarding infection risk, tampering, and errors in circuit preparation were also identified.

The survey underscores the need for standardized protocols, enhanced communication, and improved training to address the identified challenges. Recommendations include the development of clear guidelines for circuit priming, pump availability, and infection control, as well as further research in areas such as infection risk mitigation, team dynamics, and the impact of technological advancements. By implementing these recommendations, the perfusion community can strive towards enhancing patient care, reducing adverse events, and advancing perfusion safety practices.

## Abbreviations, Acronyms & Symbols

CPB: Cardiopulmonary bypass
ECMO: Extracorporeal membrane oxygenation
